# Implementation of an electronic nose for real -time identification of odour emission peaks at a wastewater treatment plant

**DOI:** 10.1016/j.heliyon.2023.e20437

**Published:** 2023-09-27

**Authors:** Stefano Prudenza, Carmen Bax, Laura Capelli

**Affiliations:** Politecnico di Milano, Department of Chemistry Materials and Chemical Engineering “G. Natta”, Piazza Leonardo da Vinci 32, 20133, Milano, Italy

## Abstract

This paper proposes a novel approach for the real-time monitoring of odour emissions from a WasteWater Treatment Plant (WWTP) using an Instrumental Odour Monitoring System (IOMS). The plant is characterized by unpredictable odour peaks at its arrival tank (AT), generating nuisance and complaints in the population living nearby the plant. Odour peaks are most likely due to the conferment of non-identified and malodorous wastewaters coming from various industrial activities. Due to the high variability of sources collecting their wastewaters to the WWTP, a new methodology to train the IOMS, based on the use of a one-class classifier (OCC), has been exploited. The OCC enables to detect deviations from a “Normal Operating Region” (NOR), defined as to include odour concentrations levels unlikely to cause nuisance in the citizenship. Such deviations from the NOR thus should be representative of the odour peaks. The results obtained prove that the IOMS is able to detect real-time alterations of odour emissions from the AT with an accuracy on independent validation data of about 90% (CI_95%_ 55–100%). This ability of detecting anomalous conditions at the AT of the WWTP allowed the targeted withdrawal of liquid and gas samples in correspondence of the odour peaks, then subjected to further analyses that in turn enabled to investigate their origin and take proper counteractions to mitigate the WWTP odour impact.

## 1 Introduction

Despite not being directly associated to risks for human health, odours are often the cause of annoyance, adversely affecting the wellness of citizens [1]. For this reason, they are, after noise, the second cause of citizens’ complaints to local authorities [2]. Moreover, different effects of the prolonged exposure to odours, including emotional stress, anxiety, discomfort, headaches, depression, eye and respiratory irritation, nausea, and vomiting, have been reported in the literature [3]. As a consequence, odour emissions have started to be subjected to control and regulation worldwide [4], which mostly involve the definition of emission limits or odour impact criteria [5,6]. This justifies the big effort done by the scientific community to study and develop suitable methods for measuring and sampling environmental odour emissions, which is also proven by the increasing number of publications concerning odour pollution over the last 40 years [7].

An interesting and promising perspective for monitoring odour emissions from industrial activities is related to the use of electronic noses (EN), more generically defined as Instrumental Odour Monitoring Systems (IOMS), individually or in networks, for a real-time monitoring of odours in the field [8]. Indeed, the odour fingerprint provided by EN (i.e., multivariate response of the gas sensor array) can be processed to provide a qualitative and quantitative characterization of odours they are exposed to. [9,10].

Over the years, thanks to this characteristic, the EN technology has been applied to the environmental monitoring of different emitting activities, such as landfills [11,12], poultry farms [13,14], oil & gas plants [15,16] and many others [17,18]. They have been successfully implemented also in WasteWater Treatment Plants (WWTP) [[19], [20], [21], [22], [23]], where the presence of multiple and extended wastewater tanks is usually associated to the emissions of unpleasant odours into the atmosphere [24]. The characterization of odour emissions from WWTP is quite challenging because they usually are complex gaseous mixtures, which can be found at very low concentrations in ambient conditions and exhibit a great variability over time in terms of both composition and concentration, mainly related to weather conditions and changing effluent load characteristics [24]. In this context, the use of an EN, being relatively low-cost and capable to operate continuously, offers several advantages compared to other techniques for odour measurement, such as dynamic olfactometry (standardized in Europe since 2003) and chemical analysis, which are more expensive and discontinuous. Capelli et al. [25] proposed the use of an EN for assessing the odour impact of a WWTP at specific receptors located in the proximity of the plant (i.e., directly where the presence of odours attributed to the WWTP were reported by citizens) through the continuous analysis of ambient air over one month. Moreover, the use of array of sensors instead of specific detectors, providing only the concentration of target gases in the odour mixture (hardly identifiable for WWTP emissions [24]), enables the characterization of the odour mixture as a whole. This can be interpreted to identify the most problematic emission sources of the plant [25,26], thereby guiding maintenance or upgrading interventions. EN have also been used to real-time assess the odour concentration at plant fenceline or at emission sources to verify the compliance with regulatory thresholds [27,28]. Innovative EN implementations, such as installation on drones, are also emerging in recent years [29,30]. Burges et al. [31,32] proved the possibility to map the odour concentration over WWTP emission sources by means of a small drone equipped with an array of electrochemical and metal oxide semiconductor sensors. The combination of drone detections with other measurement techniques could lead to the development of novel integrated approaches for odour monitoring.

Other studies exploited the use of ENs for quality and process monitoring. Wang et al. [33] focuses on investigating the correlations between IOMS signals when exposed to the headspace of liquid samples collected at the WWTP with microbiological parameters and odours emitted from different WWTP sections. Moreover, Moufid et al. [34], starting from liquid samples collected at different stages of the WWTP, developed a methodology for correlating the EN signals, when exposed to their headspace, with the anomalous presence of high quantities of pollutants. Despite their interesting and encouraging results, one of the main limitations of these studies is that the EN analyses were executed in an offline mode, lacking the investigation of the possibility to use the EN in the field for the real-time identification of anomalies in the process, which would in turn enable prompt counteraction.

In this context, this paper describes the research activity conducted at a WWTP aimed at realizing a monitoring system for continuously characterizing odour emissions from the arrival tank (AT) of the plant, which was identified as the main responsible for the odour nuisance in near-living population.

Odour concentration values exceeding 100′000 ou_E_/m^3^, which are way beyond the typical ones expected at the inlet of a WWTP, were measured in some olfactometric campaigns carried out at the plant for the preliminary characterization of its odour emissions. Such anomalies, occurring in an unexplainable manner, were supposed to be caused by the conferment of particularly odorous wastewaters, whose origins were hardly identifiable.

In order to investigate the causes of anomalous odour peaks, the monitoring system, based on EN technology, was trained to detect their occurrence at the AT and produce an alarm. The alarm contextually activated an automatic gas sampler for the targeted collection of liquid and gas samples to be characterized by olfactometric and chemical analyses. Such analyses aimed at identifying the major malodorous compounds responsible of the high odour loads and possibly determining their provenance by comparison with the chemical composition of various industrial wastewaters conferred to the WWTP to be collected upstream of the plant.

The novelty of this research concerned the approach used for the EN training. This approach involves the implementation of a one-class classifier (OCC) model to detect deviations (i.e., anomalous odour peaks) from a reference condition, referred as Normal Operating Region (NOR) [35], which is representative of moderate odour concentrations (Cod) unlikely to cause nuisance in surrounding territories. Even though the use of ENs for the real-time measurement of odour concentration in WWTPs is not new in the scientific literature, the difference here relies in the impossibility to use “traditional” regression approaches, since the high variability in terms of chemical composition of the wastewaters entering the plant results in continuously variable odour classes. Because of the unpredictability and the numerosity of the different conditions, a proper and exhaustive characterization of all possible odour classes was deemed unfeasible from both a practical and economical point of view. Thus, we had to deal with the issue of implementing a model robust and flexible enough to comprehend such different conditions and identifying anomalies without applying a regression approach.

## 2 Material and methods

### 2.1 Description of the site and related odour problems

The WWTP involved in this study is located in a mixed urban/industrial area in the North of Italy. It collects and treats both civil and industrial effluents, resulting in an incoming flowrate of about 20′000 m^3^/d. The residence time of the wastewater inside the plant is approximately 24 h. Civil wastewaters account for about 60–70% of the incoming flowrate, while approximately 30–40% comes from about 40 industrial sites located nearby the WWTP (e.g., chemical industries, printing houses and still mills).

The WWTP has a traditional configuration for biological treatment plants, as schematically illustrated in Fig. 2: It includes following units: 1) arrival tank (AT), 2) grilling, 3) sand trap/oil separator, 4) primary settler, 5) oxidation/denitrification, 6) secondary settler, 7) disinfection, 8-9) sludge treatments comprising thickening and dehydration. Except of disinfection and secondary settler, all WWTP sections are covered or located inside closed sheds, equipped with ventilators collecting exhaust air from diverse sections of the plant to two scrubbers.

**Fig. 1:**
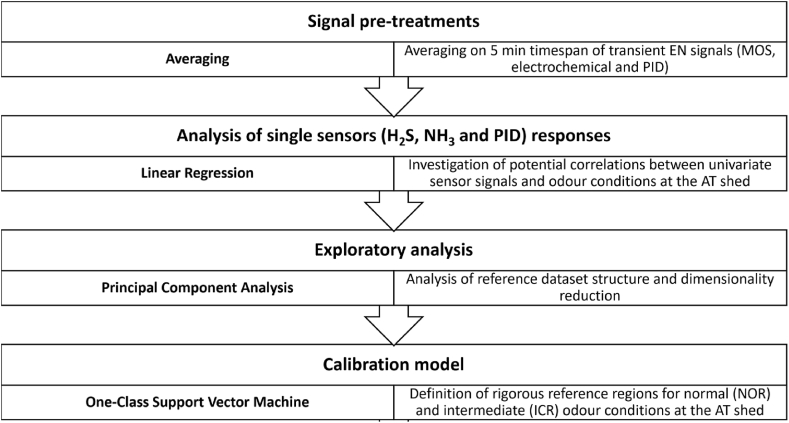
Block diagram of the data processing pipeline for calibration model development.

**Fig. 2:**
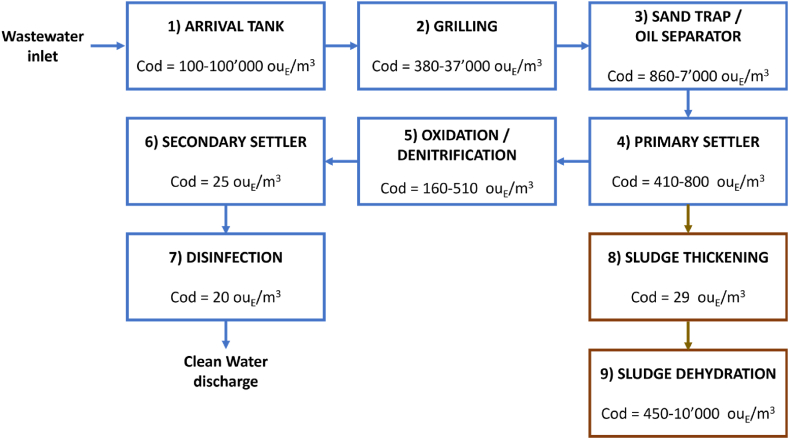
Block scheme of the WWTP: wastewater treatment line is in blue, while the sludge treatment line in brow.

Even though this plant configuration is intended to prevent odour emissions to the atmosphere, at the time of the study, some parts of the plant required maintenance and some sheds (including the one of the AT) were partially damaged, thereby resulting in unwanted and potentially annoying diffuse emissions, especially under unpredictable operating conditions. Indeed, the plant experienced a long history of odour complaints from the people living in the surroundings.

This situation has led to the execution of many investigations (i.e., olfactometric campaigns and odour dispersion modelling studies) aimed at assessing the odour impact of the plant and identifying the causes of the odour nuisance.

In the blocks indicating the different treatment units illustrated in Fig. 2, we reported also the range of the odour concentration values measured at the different WWTP sections.

As expected, the primary treatments are those associated with the highest odour emission levels. On the other hand, an unusually high variability of the odour concentration associated with some sections (in some cases of 3 orders of magnitude) can be noticed.

More in detail, the odour concentrations measured at the AT ranged from about 100 to over 100′000 ou_E_/m^3^, pointing out an extremely high variability of the odour emissions, which resulted to be uncorrelated with specific meteorological or plant operating conditions. Therefore, it was hypothesized that such peaks should be more likely linked to the conferment of particularly odorous wastewaters, causing a sudden increase of the odour concentration up to anomalous levels (around or even above 100′000 ou_E_/m^3^). Because of the number and variability of the industrial effluents entering the WWTP, together with the impossibility to get information about the exact conferment time, the occurrence of odour events resulted unpredictable, and thus their origin unknown.

One possible way to investigate the causes of the odour problem would require the collection of liquid and gas samples at the AT concurrently with anomalous odour peaks and their characterization in terms of odour concentration and chemical composition to identify the molecules that are most responsible for the odour load of the wastewater. Such investigation may lead to the identification of the industrial discharges mostly responsible for the anomalous odour emissions.

However, as previously mentioned, the occurrence of the odour peaks is unforeseeable and not very frequent. Therefore, the execution of random samplings would have resulted in a huge number of potentially useless analyses of samples collected outside of odour peaks, further resulting in unsustainable costs. For these reasons, we thought about the possibility to realize a dedicated monitoring system, based on the EN technology, able to real-time detect the occurrence of the odour peaks at the AT, and thus allowing the withdrawal of samples in a targeted manner, i.e., only in correspondence of anomalous events.

### 2.2 Electronic nose and sampling system

For this study, we used an EN WT1 commercialized by Ellona, an automatic gas sampler (Scentroid, model VC20) and an automatic liquid sampler (Glacier, model 6700) for the targeted withdrawal of gaseous and liquid samples for olfactometric and chemical characterization.

The WT1 is a commercial EN for outdoor use, which can be equipped with different sensors according to the specific application. In this case, the choice of the EN array configuration was defined based on a preliminary evaluation of the expected wastewaters composition, which was based on a preliminary study of the technical documents and environmental permits of the major industries discharging to the WWTP under examination.

The WT1 was equipped with the following sensors.:

• 2 non-specific metal oxide sensors (MOS), characterized by a high sensitivity to Volatile Organic Compounds (VOC);
• 3 specific electrochemical sensors for the detection of hydrogen sulphide (H_2_S), formaldehyde (CH_2_O) and ammonia (NH_3_);
• 1 photoionization detector (PID) calibrated in isobutylene, for estimating the total VOC concentration in ppm.

Table 1 reports the limit of detection (LOD), range and resolution of the electrochemical and PID sensors mounted in the WT1.

**Table 1:** Limit of Detection (LOD), range and resolution of the electrochemical and PID sensors mounted in the WT1.

Sensor	LOD [ppm]	Range [ppm]	Resolution [ppm]
Formaldehyde	0.002	0–20	0.001
Ammonia	0.09	0–100	0.03
Hydrogen sulphide	0.015	0–12	0.005
PID	0.005	0–20	0.01

The instrument comprises also four sensors for measuring the temperature and relative humidity of the sensor chamber and of the external environment. Furthermore, the EN is equipped with a SIM card for data storage in cloud and users’ remote connection through the access to a specific web-platform.

**Fig. 3:**
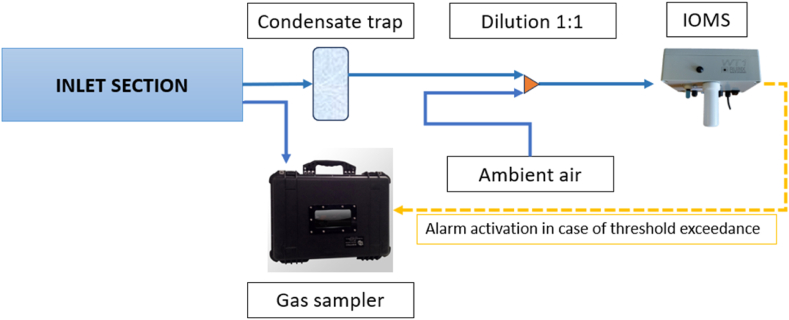
Schematic representation of the sampling system implemented at the AT of the WWTP for both EN analysis and samples withdrawal.

The EN has a suction rate of approximately 1 L/min and provides responses at a frequency of 0.1 Hz. Given the high levels of hydrogen sulphide and humidity expected at the AT, the EN sampling system was appositely modified for the specific application, with the purpose of ensuring a 50% dilution of the air sucked from inside the AT shed with external air. Indeed, sensors' exposure to extreme conditions may damage the EN hardware and accelerate sensor drift [36]. The dilution system consists of a ‘Y’ connector, which connects two Teflon tubes sucking external ambient air and air from inside the AT shed, respectively (Fig. 3).

The automatic gas sampler consists of an airtight suitcase of 25 L allowing the filling of a 6 L Nalophan™ bag by means of a membrane vacuum pump, sucking air from the AT shed through a Teflon tube. The collection of the gas sample lasts about 1 min. The sampler can be activated manually or through a digital signal, which can be sent by the remote platform or by the EN to which it is connected when specific alarm thresholds are exceeded.

**Fig. 4:**
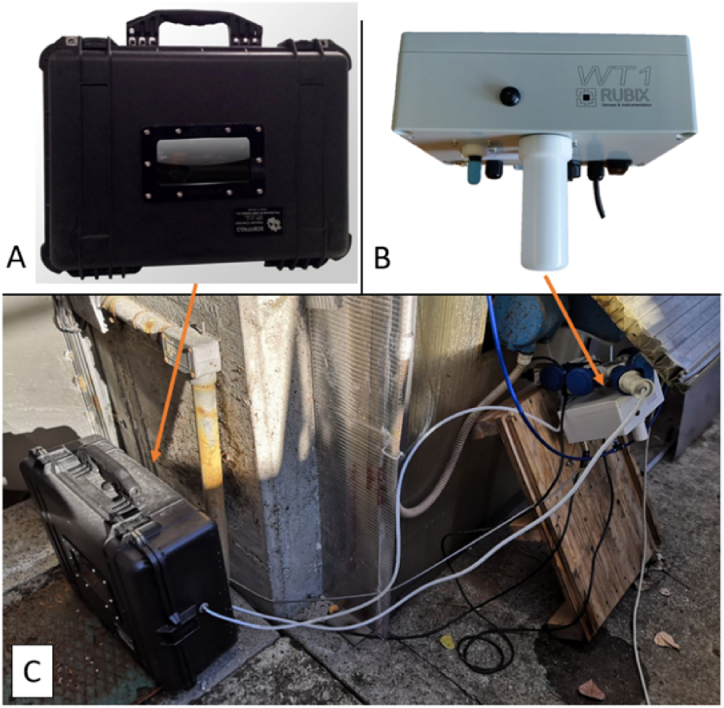
The sampling chamber (A) and the WT1 (B) used in this study installed on the arrival tank of the WWTP (C).

Fig. 4 illustrates pictures of the IOMS (Fig. 4 B) and the automatic gas sampler (Fig. 4 A), and their installation at the WWTP outside of the AT shed (Fig. 4 C).

### 2.3 IOMS training

#### 2.3.1 Traditional approach and novel proposal

The IOMS training represents a crucial phase for environmental odour monitoring. Training consists in the creation of a dataset including samples having the characteristic “patterns” of the odours that the instrument is expected to be exposed to during the monitoring phase [37]. It is worthy highlighting that an inappropriate training might compromise the EN performance and result in misclassifications or under/over-estimations of the concentrations of the odours detected [38,39].

In general, the IOMS training involves the execution of olfactometric campaigns at the most significant odour emission sources of the plant(s) under investigation and the preparation of suitable odour samples to be presented to the EN for implementing training models. Depending on the goal of the monitoring, training models could be based on classification (i.e., qualification of the ambient air) or regression (i.e., quantification of the odour concentration) approaches [37].

Static and dynamic calibration protocols have been proposed in the scientific literature as methods for training an EN to recognize and/or quantify odour emissions. The static calibration consists in presenting to the EN bags filled with gas sampled at the emissions under study at different concentrations in alternation to fresh air. Each analysis consists of three phases: 1) baseline - the EN array is exposed to fresh air, 2) sample analysis – the odour samples is analysed for a few minutes, 3) recovery - the signal baseline is restored with fresh air. The static response thus acquired is then correlated with the odour class and/or concentration of the sample by applying machine learning techniques for classification or quantification purposes [29].

Conversely, dynamic calibration involves the acquisition and processing of continuous EN signals acquired when the instrument is directly exposed to the odour emissions under exam. The dynamic calibration has the advantage of training the instrument in the same way it will be operated during the monitoring phase, when it will be installed at the desired site (directly at the emission source, plant fenceline or the receptor) to provide a continuous qualitative/quantitative characterization of the air [29]. In this case, the information about the odour type or concentration relies on the characterization by dynamic olfactometry or chemical analysis of samples collected at the odour source in some specific moments (e.g., particular operating condition of the monitored process, production of different products, etc).

For the specific monitoring here described, traditional calibration approaches are not applicable as such. As already mentioned in section 3.1, the wide range of possible odour conditions occurring at the AT, associated to the high number and types of incoming effluents, makes traditional training unfeasible. Indeed, building an accurate classification model to recognize all the different combinations of different wastewaters entering the plant would require the analysis of an enormous number of samples, resulting in an extremely expensive process in terms of both costs and duration.

As a consequence, also the development of a regression model capable of estimating the odour concentration at the AT was deemed inapplicable. Different types and combinations of wastewaters, characterized by different chemical compositions, even if characterized by similar odour concentrations may generate very different EN sensor responses, and vice versa. This would introduce unacceptable bias in the EN regression model and lead to major errors in the estimations of the odour concentrations.

To overcome such limitations, we decided to try a different and novel approach. It is based the implementation of a one-class classification model for the real-time detection of deviations (i.e., anomalous odour peaks) from a refence region representative of “normal” odour conditions at the AT of the WWTP, referred as Normal Operating Region (NOR) [40]. To do this, a reference dataset, including EN odour fingerprints of the ambient air in the AT shed representative of sufficiently low odour concentrations - unlike to cause nuisance outside the plant - was defined and used to implement the training model, as will be deeply described in section 1.1.

The proposed approach differs from conventional multi-classification problems since, for implementing the training model, it requires only the definition of the reference condition, without needing a detailed representation of all the scenarios (i.e., different wastewater types and combinations thereof) possibly occurring during the monitoring. One-class algorithms have already been used for several applications [[41], [42], [43], [44], [45], [46]], but, to the best of our knowledge, it is the first time that they are implemented for environmental odour monitoring with an EN.

#### 2.3.2 Data acquisition

During the training phase, the EN was installed at the WWTP to continuously analyse the ambient air in the AT shed and different olfactometric measurement were carried out according to EN 13725:2022 with the purpose of getting the information about the odour concentration levels, which are needed for implementing training models.

Olfactometric measurements were carried out randomly during the training phase, which lasted about 30 days, with the purpose of capturing the monthly variability of the WWTP odour emissions, as had been pointed out by the previously mentioned preliminary olfactometric studies. A total of 18 gas samples were collected on 10 different days and at different hours (i.e., early morning, midday, afternoon, and night), under different meteorological conditions (i.e., sunny, foggy, and rainy days) and various operating conditions of the WWTP (i.e., different inlet flowrates and presence/absence of sludge recirculation).

This allowed also investigating the effects on EN signals of the daily variations of temperature and humidity levels, which are known as potential interferents from the literature [47]. During the training, temperature in the AT oscillated between 14 °C and 23 °C, with a mean value of about 20 °C. Conversely, relative humidity ranged from 20% to 40% with a mean value close to 40%. Such oscillations did not affect EN signals stability over the monitoring and had negligible effects on the EN capability to detect critical odour conditions, as reported in the following.

#### 2.3.3 Data processing

Fig. 1 schematically describes the data processing pipeline applied to implement the calibration model for the EN monitoring. RStudio was used for data processing (RStudio Team (2020). RStudio: Integrated Development for R. RStudio, PBC, Boston, MA URL http://www.rstudio.com/).

##### 2.3.3.1 Signal pre-treatments

The training dataset was built considering transient EN signals acquired concurrently with the sample withdrawal, lasting about 1 min, in combination with results of the olfactometric analyses carried out on gas samples randomly collected at the AT shed during the training phase.

More in detail, transient EN signals recorded by the 6-sensors array in the 5 min comprising sample withdrawal (i.e., 2 min before + 1 min sampling + 2 min after) were averaged and converted into multidimensional vectors, whose components, usually referred to as features, were the raw resistance values (in Ohm) recorded by the MOS sensors and the analytical concentrations (in ppm) provided by electrochemical and PID sensors. 5-Min averaging was applied to all the sensors of the EN array for eliminating noise and disturbances from raw sensors data, which could interfere with further processing. The timespan of 5 min resulted to be effective in removing potential disturbances without affecting relevant information about the correlation with the odour events at the AT, which have a considerably longer duration (i.e., from 30 min to 2–3 h). Thus, the training dataset resulted in an 8219 × 6 data frame, comprising 8219 observations (1 observation for every 5 min of signal acquisition during the training phase) and 6 features (averaged responses of the 6 EN sensors).

##### 2.3.3.2 Analysis of single sensors’ responses

Before constructing multivariate calibration models based on the responses of the whole EN sensor array, the possibility to identify a correlation between the responses of specific sensors (electrochemical sensor for H2S and PID sensor) and the odour conditions at the AT was investigated.

The hydrogen sulphide (H_2_S) sensor was selected for this analysis because this compound has been often reported in scientific literature as a tracer of odour emissions from WWTP [48,49]. The PID sensor's response was also evaluated since PID sensors are commercially proposed as a cheaper alternative to multi-sensor systems to provide information about the concentration of odour emissions in some emission scenarios [50,51].

This analysis involved the critical evaluation of responses provided by single sensors and the use of a linear regression to fit the analytical concentrations (in ppm) provided by sensors with the odour concentration obtained by dynamic olfactometry. The coefficient of determination R^2^ was used as an indicator for the goodness of the regression [52].

##### 2.3.3.3 Exploratory analysis

Principal Component Analysis (PCA) [53] was selected for reducing data dimensionality and performing a preliminary investigation of their structure aimed at evaluating the existence of a correlation with odour conditions at the AT shed.

This technique projects the original data into a new reference system, whose components, named Principal Components (PC), which are orthogonal and independent among them, represent the directions of the maximum variance of the data [53]. The power of this methodology is that, in most cases, the use of the first 2 or 3 PCs is sufficient to capture most of the variance of the original dataset. Therefore, PCA allows visualizing the data in a 2D/3D graph, the so-called “score plot” [53]. Moreover, the PCA “loading plot” [54] provides information about the correlation of features extracted from sensors’ responses and on their contribution to samples discrimination. Thus, combining PCA outputs enables the interpretation of data structure, making similarities or differences among different groups of observations explicit, and at the same time understanding which are the variables that mostly affect these groupings [54].

Being an unsupervised technique, PCA does not require information about the belonging class of the observations, but it groups them in the new space on the basis of common characteristics. This peculiarity was particular useful for this specific monitoring scenario, which, as already mentioned, is characterized by the impossibility to define a priori a set of different odour classes.

##### 2.3.3.4 Definition of the Normal Operating Region (NOR)

Considering the final goal of the research, which is the real-time identification of anomalous odour peaks at the AT of the WWTP under investigation, the data processing procedure focused on the definition of a Normal Operating Region (NOR) representative of normal odour conditions at the plant. This first required the definition of an upper limit for the odour concentrations detectable at the AT unlikely to cause odour nuisance outside the WWTP. For these evaluations, we used the results of odour dispersion models implemented on previous olfactometric campaigns carried out at the WWTP. Specifically, odour dispersion models investigated the correlation between the odour concentration inside the AT shed and the odour impact at closest receptors considering the meteorological conditions and orography of the site under study. The results of such studies pointed out that odour concentrations above 5′000 ou_E_/m^3^ at the AT could cause - under specific meteorological conditions that are quite frequent in the area - significant odour events in the surroundings of the plant. Therefore, gas samples collected during the training phase having an odour concentration below 5′000 ou_E_/m^3^ were used to define the reference odour dataset. The reference odour dataset comprises 7′067 observations out of the total 8219 acquired during the training and Cod values ranging from 140 to 5′000 ou_E_/m^3^.

This reference odour dataset satisfied following conditions on the PCA scores plot obtained considering the entire training dataset.

1. PC1 values higher than 0 (PC1 > 0 ^ ∀ PC2),
2. PC1 values comprised between −2.5 and 0, and PC2 values comprised between −3 and 2 (-2.5 < PC1 < 0 ˄ −3 < PC2 < 2).

The scores obtained for the reference odour dataset were used as input of a One-Class Classifier (OCC) for defining in a rigorous way the NOR boundary. Among the different OCC algorithms suggested in literature, the one-class Support Vector Machine (one-class SVM) developed by Sholkopf et al. [55] was selected. This algorithm, like common “multi-class” SVM, constructs a function (or hyperplane) around the “reference” data provided, with the purpose of defining a “belonging region” for such class [56].

In this work, a gaussian kernel function was chosen for implementing the calibration model, since it allows also considering the information about the density distribution into the space of the training data for boundary definition. The boundaries of the NOR region can then be properly shaped by modifying some specific input parameters of the model: *nu* and *gamma*. The *nu* parameter can be interpreted as the probability of finding a new regular train observation outside the boundary defined by the model [57], while the *gamma* parameter provides information about how much each single point influences boundaries shape [58]. In general, such parameters are optimized through cross validation approaches. Because of the limited and unbalanced reference odour dataset due to above-mentioned limitations in foreseeing different odour conditions, *nu* and *gamma* were varied manually and selected to avoid over-fitting on training samples. In this way, also odour conditions not sampled during the training but expected to result in low odour concentrations based on PCA score plot (see Fig. 9) were included. For the specific monitoring, *nu* and *gamma* were stetted to 0.0001 and 0.03, respectively.

##### 2.3.3.5 Definition of an Intermediate Odour Region (IOR)

Due to the high variability of the odour concentrations spotted at the AT both during this study and in previous olfactometric campaigns, two odour regions were defined to further classify the odour conditions falling outside the NOR, which were referred as *intermediate* and *critical* regions.

The *intermediate* region comprises odour events having a Cod between 5′000 ou_E_/m^3^ and 10′000 ou_E_/m^3^, corresponding to the NOR limit and its double, respectively. Conversely, the *critical* region includes odour events with Cod above 10′000 ou_E_/m^3^, which are very likely to produce a considerable odour impact on the surroundings of the WWTP, generally resulting in an increase of citizens' complaints.

With the aim to develop a model for promptly differentiating critical odour events, a second one-class radial SVM was implemented on an extended reference odour dataset to define an Intermediate Odour Region (IOR). This extended reference odour dataset included training samples with odour concentrations up to 10′000 ou_E_/m^3^, resulting in a data frame of 7′700 observations out of the total 8219 acquired during the training. Such samples satisfied following condition on the PCA scores plot obtained considering the entire training dataset.

1. PC1 values higher than 0 (PC1 > 0 ^ ∀ PC2), PC1 values comprised between −4 and 0, and PC2 values comprised between −4 and 3 (-4 < PC1 < 0 ˄ −4 < PC2 < 3).

In this case, the one-class SVM model, based on a gaussian kernel function, was built by fixing nu and gamma parameters at 0.0001 and 0.03, respectively.

### 2.4 Monitoring phase and validation of calibration models

After training, the EN continuously analysed the ambient air in the AT shed and provided a real-time characterization. The monitoring phase lasted about two months, resulting in a monitoring dataset of 15765 observations (i.e., data averaged on 5 min timespan).

In this phase, 10 independent gas samples were collected by the automatic sampler and used for validating the calibration models. They were sampled both in case of alarm threshold exceedance and randomly under normal conditions with the purpose of creating a relatively equally-distributed validation dataset. These validation samples were then analysed by dynamic olfactometry to determine their odour concentration and compare the obtained results with the predictions made by the EN to evaluate, in an unbiased way, the real performances of the system [59].

Monitoring data were processed in real-time according to the data processing pipeline above described. In case of exceedance of NOR boundaries, the EN reported an alarm and contextually activated the automatic sampler for collecting the gaseous samples to be used for further investigations aimed at identifying the causes of anomalous odour peaks at the WWTP (i.e., execution of targeted olfactometric and chemical characterizations). Moreover, the frequency of the occurrence of the odour peaks, as reported by the EN, was analysed, with the purpose of investigating possible trends in the occurrence of those anomalies’, as for instance identifying critical hours of the day or days of the week during which they happened more frequently.

## 3 Results and discussion

### 3.1 Analysis of single sensors responses

**Fig. 5:**
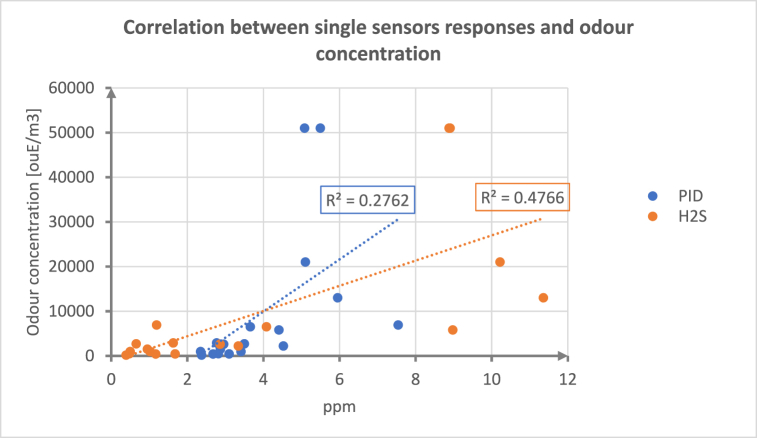
Evaluation of the correlation between the PID and H_2_S sensors responses [in ppm] and the odour concentration values [in ou_E_/m^3^] of the samples taken from the arrival tank during the training phase measured by dynamic olfactometry.

Fig. 5 compares the univariate response of H_2_S and PID sensors with the odour concentration of the samples withdrawn at the AT and assessed by dynamic olfactometry. The plot highlights a very poor correlation, with R^2^ values equal to 0.28 and 0.48 for the PID and the H_2_S sensors, respectively.

Based on this evaluation, the use of single gas sensors (H_2_S and PID) was proven unsuitable for a real-time characterization of the entity of the odour emissions from the investigated source. This further proves the need to use multivariate approaches capable of providing a characterization of the odour emissions as a whole instead of focusing on tracers. Besides being in many cases hardly identifiable, tracers can only provide partial information about the odour emission. Moreover, the responses of specific analysers are often affected by interferences due to the multiple presence of different chemical compounds, as it is for instance the case of WWTP emissions, whose composition considerably changes over the monitoring period due to meteorological conditions and WWTP operating conditions [60]. Furthermore, it is also well established that simply summing Odour Activity Values (OAV) relevant to compounds present in an odour mixture is not always enough to obtain a correct estimation of its odour concentration: due to the so-called synergism phenomena among different molecules the overall odour load could result lower or higher than as expected [61,62]. Therefore, except of in the case of very simple mixtures (which is not the case of WWTP emissions), the approach of looking at a single compound or at a single sensor response is oversimplified and does not produce reliable results in terms of correlation with the odour concentration, as already discussed in the scientific literature [63].

### 3.2 Exploratory analysis

**Fig. 6:**
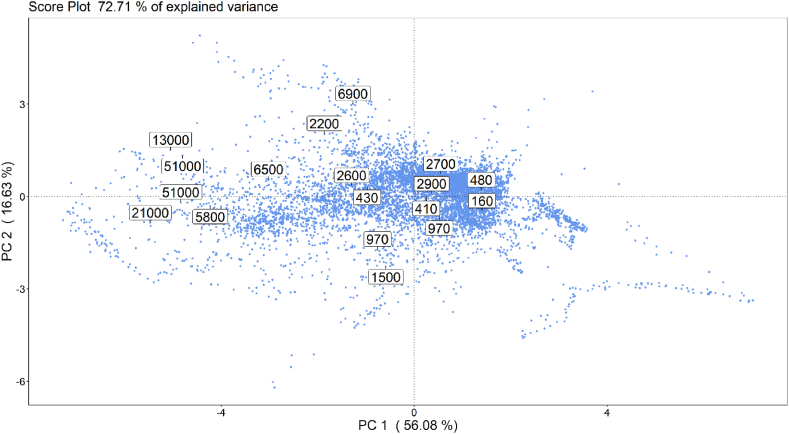
PCA scores plot obtained on the training data: numbers displayed indicate the odour concentration, expressed in ou_E_/m^3^, of the 18 gas samples collected at the arrival tank during the training phase.

**Fig. 7:**
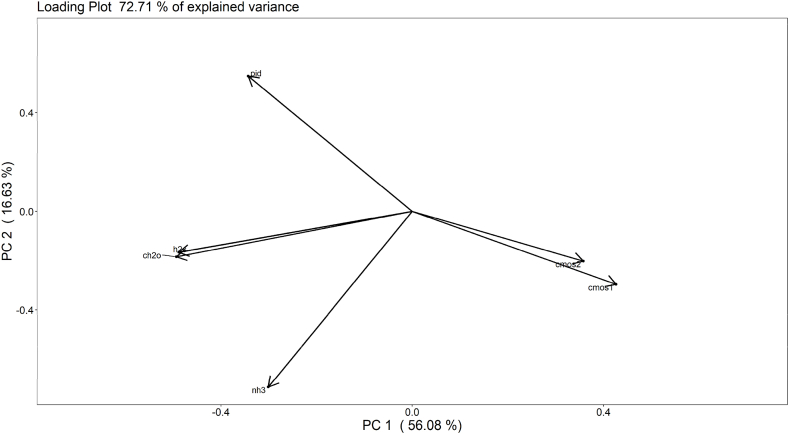
PCA loading plot obtained on the training data: the acronyms “cmos1” and “cmos2” stand for the 2 MOX sensors, “pid” stands for the PID sensor, while “h2s”, “ch2o” and “nh3”, stand for the H_2_S, CH_2_O and NH_3_ electrochemical sensors, respectively.

As described in Section 2.3.3, data collected during the training phase were processed by PCA, with the purpose of reducing their dimensionality and investigating their structure. Based on the visual inspection of the scree plot, illustrating the explained variance associated to various PC, a 2-D PCA model, describing about 72.7% of the dataset variance, was implemented. The PCA scores and loading plots obtained on the training dataset are reported in Fig. 6, Fig. 7, respectively.

The score plot (Fig. 6), illustrating the projection of observations into the new reference system defined by the first two PC, points out the existence of a correlation between training samples' distribution along the PC1 and the odour concentration measured at the AT of the WWTP (which are indicated by the labels reported in the plot). More in detail, odour concentration values tend to decrease along the PC1 axis. Thus, two macro-clusters could be defined based on the odour concentration values: samples with Cod lower than 5′000 ou_E_/m^3^ (which is considered as the limit value below which it is unlikely that odour nuisance occurs outside the plant) clustered in the right portion of the PCA score plot, while samples with Cod above 5′000 ou_E_/m^3^ are distributed in the left portion of the PCA score plot.

For a deeper insight of the PCA results, the loading plot (Fig. 7) was evaluated. It highlights that the left region of the score plot, where most of the training samples having high Cod cluster, is characterized by increased concentrations (in ppm) of H_2_S and CH_2_O measured by electrochemical sensors, and decreased MOS electrical resistances (as expected for n-type sensors if exposed to VOCs). This points out that H_2_S, CH_2_O and MOS sensors responses together most contribute to provide information about the odour conditions at the AT. Conversely, the contribution of the PID and NH_3_ sensors in expressing odour concentrations is less significant compared to the other sensors of the array.

Concerning the PID sensor, this result confirms what obtained by the univariate analysis of its responses, discussed in section 3.1. On the other hand, considering the NH_3_ sensor, its poor correlation is probably due to the low levels of ammonia registered on the AT, varying between 0.3 and 1.5 ppm, which are negligible compared to the higher values measured by the other electrochemical sensors, which are in the order of 1–10 ppm.

**Fig. 8:**
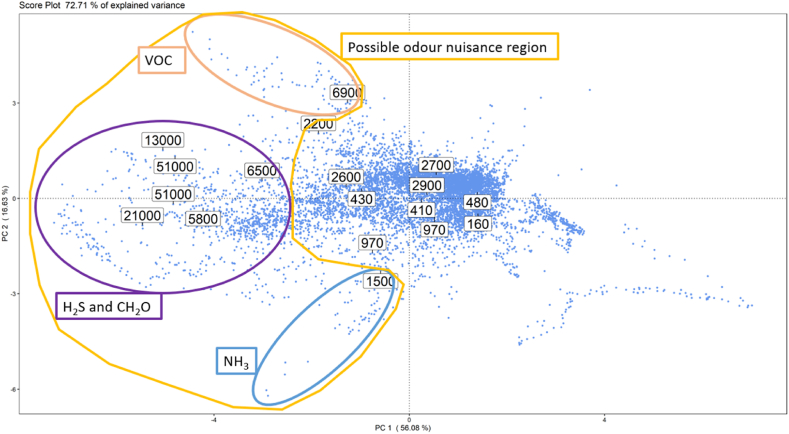
Visual representation of the 3 main “regions of odour” that can be identified on the PCA score plot.

Finally, combining the information provided by the score and loading plots, and looking at the distribution of the scores along the PC2, it is possible to identify 3 main “regions of odours” on the score plot: one in the top-left, one in the middle-left and one in the bottom-left part of the score plot (Fig. 8). These 3 regions are associated to different odour conditions at the AT, associated to high levels of VOC, of hydrogen sulphide and formaldehyde, and of ammonia, respectively. This means that when a new point is projected onto one of these 3 regions it is likely that an anomalous odour peak is occurring on the arrival tank, caused respectively by one of those families of components.

**Fig. 9:**
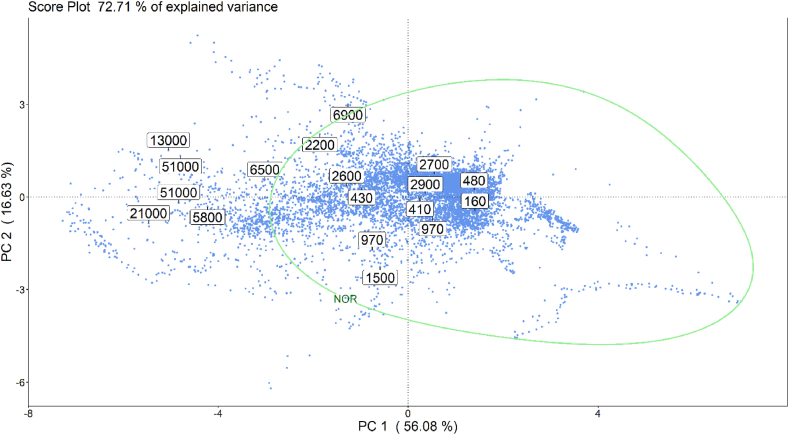
Illustration on the PCA score plot obtained on the training dataset of the NOR boundary (green line) implemented by the one class SVM model.

### 3.3 Implementation of the one-class SVM algorithm

#### 3.3.1 Normal Operating Region (NOR)

After the visual inspection of the data by PCA, a NOR was defined by implementing a one-class SVM model aimed at identifying anomalous emitting conditions the AT, which are likely to cause the perception of odours outside the plant.

Fig. 9 shows in green the boundary of the NOR region defined on the reference odour dataset. As described in section 2.3.3, the NOR comprises all training samples having an odour concentration below 5'000 ou_E_/m^3^.

#### 3.3.2 Intermediate Odour Region (IOR)

As a second step, we focused on the building of an OCC model to be used for identifying critical odour conditions at the AT of the WWTP, which may lead to odour events even at far distance from the plant.

**Fig. 10:**
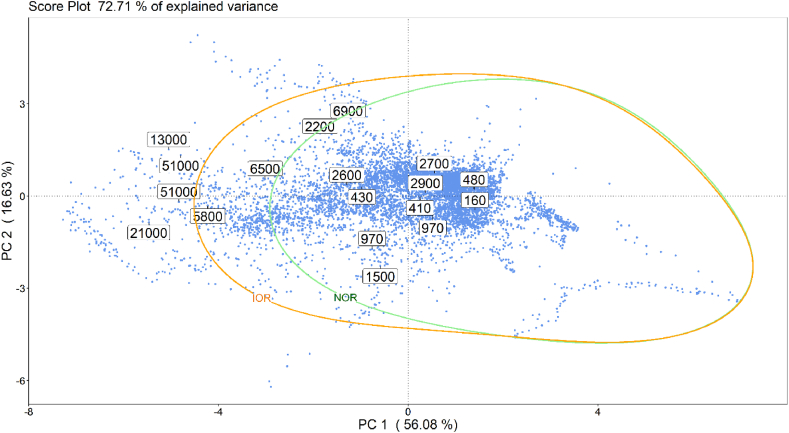
Illustration on the PCA score plot obtained on the training dataset of the NOR (green line) and the IOR (orange line) boundaries implemented by the one class SVM algorithm.

The definition of an Intermediate Odour Region was intended to differentiate odour events in terms of intensity, thereby easing the assessment of the frequency with which very critical odour conditions occurred at plant and, ideally, enabling the identification of their possible causes. As previously mentioned, the intermediate region refers to odour concentrations between 5′000 ou_E_/m^3^ and 10′000 ou_E_/m^3^. Fig. 10 illustrates on the PCA score plot obtained on the training dataset the NOR (green line) and the IOR (orange line) boundaries implemented by the one class SVM algorithm.

### 3.4 Validation of the calibration models

As described in section 3.4, after training, the EN continuously provides an output indicating the odour condition of the air withdrawn from inside the AT shed, i.e., indicating whether the analysed air belongs to the NOR, IOR or neither of the two (i.e., Cod >10′000 ou_E_/m^3^). A validation session was carried out at the WWTP during the monitoring phase to assess the EN capability to correctly detect and signal the occurrence of anomalous odour events exceeding both NOR and IOR.

The following sections report the results of the validation tests relevant to NOR and IOR models, respectively.

#### 3.4.1 Normal Operating Region (NOR)

**Fig. 11:**
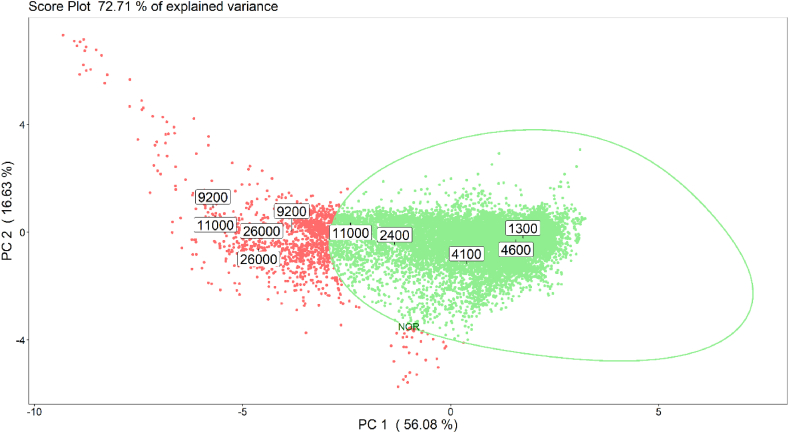
Projection of validation data on the PCA model implemented on training data, illustrating the boundary of the NOR region (green line). Point colours indicate the NOR model prediction (green = NOR, red = alarm).

Fig. 11 shows the score plot obtained by projecting the validation data acquired at the AT during the monitoring phase on the PCA model implemented on training data. Validation data are coloured according to the EN predictions based on NOR model. More in detail, points corresponding to EN measurements are coloured in green if they fall within the NOR or in red if they are classified as anomalous events, i.e., projected out of the NOR boundary. Point labels in Fig. 11 report the odour concentration values of the validation samples measured by dynamic olfactometry.

**Table 2:** Summary of validation tests aimed at evaluating the performance of the NOR model.

Odour concentration [ou_E_/m^3^] (C.I. 95%)	Expected classification	Prediction by NOR model
1'300 (570–2'500)	NOR	NOR
2'400 (1'100–4'600)	NOR	NOR
4'100 (1'800–7'900)	NOR	NOR
4'600 (2'000–8'900)	NOR	NOR
9'200 (4'100–18'000)	Alarm	Alarm
9'200 (4'100–18'000)	Alarm	Alarm
11'000 (4'800–21'000)	Alarm	NOR
11'000 (4'800–21'000)	Alarm	Alarm
26'000 (11'000–50'000)	Alarm	Alarm
26'000 (11'000–50'000)	Alarm	Alarm

Table 2 summarizes the results of the validation tests, reporting, for each validation sample, the odour concentration measured by olfactometry, the expected classification based on the odour concentration criterium defined to implement the NOR model, and the prediction by the NOR model. The odour concentration of 10 validation samples collected at the AT ranged from 1'300 to 26'000 ou_E_/m^3^. Table 3 reports the corresponding confusion matrix, resulting in a classification accuracy of 90% (CI_95%_ 55–100%).

**Table 3:** Confusion matrix relevant to the external validation of the NOR model.

		Expected classification	
		NOR	Alarm
**Prediction by NOR model**	*NOR*	4	1
	*Alarm*	0	5

Despite the relatively small number of test samples, the results of the validation tests proved the potentiality of the developed approach for the real-time detection of anomalous odour conditions at the WWTP. Indeed, the NOR defined by the model proved to be suitable for discriminating “normal” odour concentration levels at the AT, unlikely to cause odour events in the surroundings, from anomalous odour peaks. Moreover, the developed system turned out to be effective in signalling the exceedance of the critical odour concentration and activating automatic sampling systems for the collection of gas samples for further investigations.

It is important to highlight that the approach here proposed allows the identification of anomalous peaks potentially having very different compositions and consequently very different odour fingerprints, without requiring the characterization of each different odour type/class singularly for the EN training. Thus, it results particularly effective in emission scenarios characterized by high variability in terms of odour composition and concentration (e.g., different sources or operating conditions or productions).

#### 3.4.2 Intermediate odour region(IOR)

The validation of the IOR model was carried out exploiting the same methodology described above for the NOR. Table 4 compares the odour concentration and the expected classification of the 10 validation samples with the predictions provided by the IOR model (i.e., NOR, IOR or alarm), while Table 5 reports the corresponding confusion matrix. The IOR model achieved on validation data a classification accuracy of 75% (CI_95%_ 44–97%) and a recall for different classes corresponding to 100%, 50% and 75%, for NOR, IOC and alarm classes, respectively.

**Table 4:** Summary of validation tests aimed at evaluating the performance of the IOR model.

Odour concentration [ou_E_/m^3^] (C.I. 95%)	Expected classification	Prediction by IOR model
1'300 (570–2'500)	NOR	NOR
2'400 (1'100–4'600)	NOR	NOR
4'100 (1'800–7'900)	NOR	NOR
4'600 (2'000–8'900)	NOR	NOR
9'200 (4'100–18'000)	IOR	IOR
9'200 (4'100–18'000)	IOR	Alarm
11'000 (4'800–21'000)	Alarm	NOR
11'000 (4'800–21'000)	Alarm	Alarm
26'000 (11'000–50'000)	Alarm	Alarm
2'6000 (11'000–50'000)	Alarm	Alarm

**Table 5:** Confusion matrix relevant to the external validation of the IOR model.

	Expected classification		
	NOR	IOR	Alarm
EN prediction	NOR	4	0
	IOR	0	1
	Alarm	0	1

**Fig. 12:**
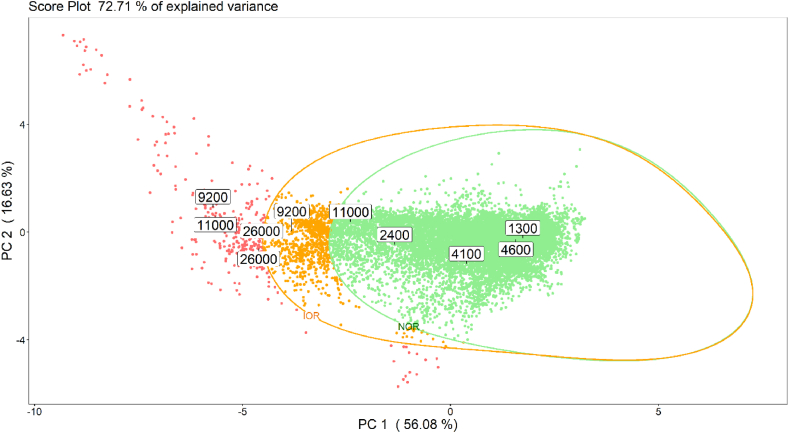
Projection of validation data on the PCA model implemented on training data, illustrating the boundaries of the NOR (green line) and IOC (orange line) regions. Points' colours indicate the IOR model prediction (green = NOR, orange = IOR, red = alarm).

The projection of validation data on the PCA model is reported in Fig. 12, whereby the points are coloured according to the IOR model predictions.

As expected, the performance of the IOR model is lower than the one achieved by the NOR model, mainly because of the poor classification performance obtained on the IOR. This result is most likely related to the small representation of the IOR class both in the training and in the validation dataset. Indeed, during the training phase, only 3 out of the 18 training samples turned out to have an odour concentration between 5′000 and 10′000 ou_E_/m^3^ occurred. Moreover, with a validation dataset comprising only 2 samples, one incorrect classification immediately results in a decrease of the classification accuracy by 50%.

Despite being less powerful than the NOR model, the IOR model offers the possibility to obtain rough information about the intensity of the odour events occurring at the WWTP, thereby discriminating “very critical” conditions from “intermediately critical” ones. Such information is very useful from a management point of view since it can guide interventions from the plant operators.

### 3.5 Monitoring results

#### 3.5.1 Analysis of NOR exceedances registered by the EN

The validated calibration models were used to process the monitoring data, and some statistical evaluations were carried out on the EN predictions to assess the frequency of odour peaks at the AT, with the purpose of possibly identifying some trends and investigating their nature.

**Fig. 13:**
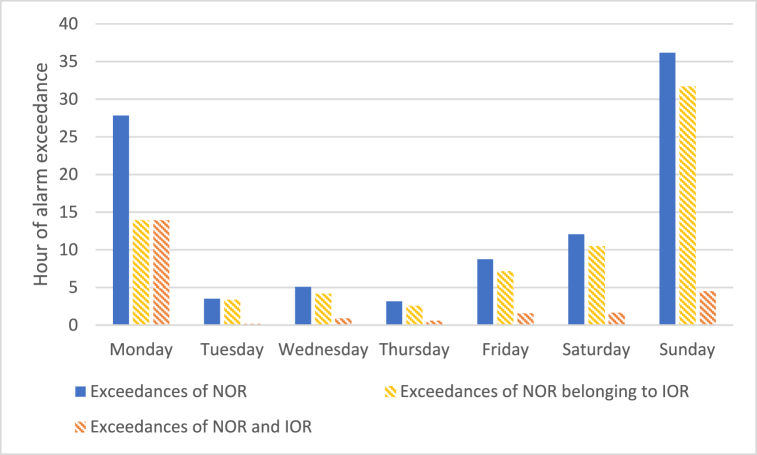
Number of hours of threshold exceedance observed for the different days of the week. The solid bars (blue) has been obtained considering only the model developed with the NOR, while dotted ones includes both NOR (yellow) and IOR (orange). The solid blue bars correspond to the sum of dotted yellow and red bars.

A first evaluation concerned the analysis of exceedances of NOR or IOR recorded on different days of the week to investigate the existence of a weekly trend for odour events. Fig. 13 reports, for each day of the week, the average number of monitoring hours during which the EN signalled the exceedance of NOR and/or IOR boundaries, i.e., the exceedance of odour concentration values of 5′000 (NOR) and 10′000 ou_E_/m^3^ (IOR), respectively. Specifically, it reports as solid blue bars the exceedances of the NOR, as dotted yellow bars the exceedance of NOR belonging to IOR, and as dashed red bars exceedances of both NOR and IOR (i.e. the sum of dotted yellow and red bars corresponds to value of solid blue bars).

The plot in Fig. 13 highlights a trend in NOR and IOR exceedances recorded on different days. Few hours of exceedances (i.e., about 5 h) were recorded in the middle of the week (i.e., on Tuesdays, Wednesdays and Thursdays), and a progressive increase of the number of NOR or IOR exceedances was registered from Fridays to Mondays (i.e., from about 5 h up to about 30 h), reaching the highest value on Sunday.

**Fig. 14:**
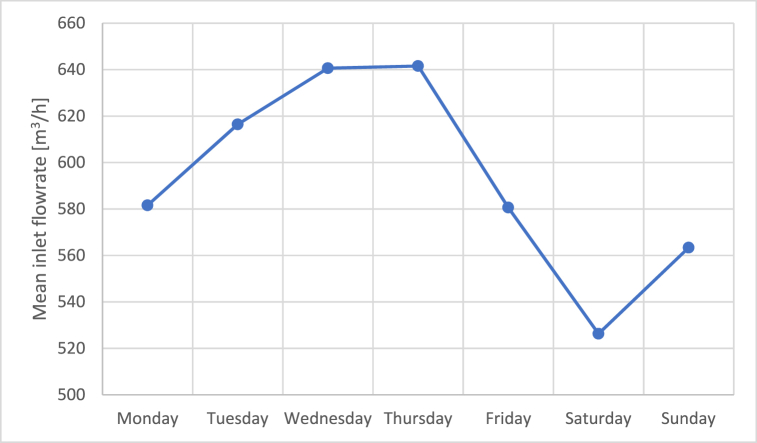
Weekly trend of the mean inlet flowrate at the WWTP.

This trend could be explained by the periodical interruption on the weekend of some smaller industrial activities discharging their effluents to the WWTP. Such smaller industrial activities are typically characterized by the discharge of relatively diluted, and consequently scarcely odorous, effluents, making that their interruption not only reduces the flowrate of the incoming wastewater to the WWTP, but it also makes it less “diluted” (i.e., more concentrated), thus generating odour emissions with increased intensity. This hypothesis is reinforced by looking at the mean inlet flowrate at the WWTP for the different days of the week registered within the monitoring period, which is reported in Fig. 14. On average, the inlet flowrate reaches its maximum in the middle of the week (i.e., about 640 m^3^/h), and decrease by about 10–20% on Fridays (Fig. 14).

Thus, these considerations may further suggest that the odorous wastewater causing anomalous odour peaks at the AT of the WWTP may come from one of the industrial activities operating 7/7 d.

**Fig. 15:**
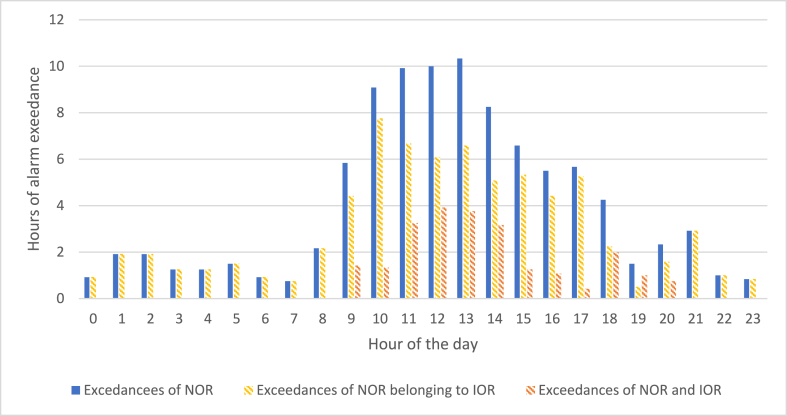
Daily trend of the number of hours exceeding the NOR.

A similar analysis was carried out on monitoring data by differentiating the hours of the day with the purpose of identifying the time slots at which odour peaks mainly occur (Fig. 15).

**Fig. 16:**
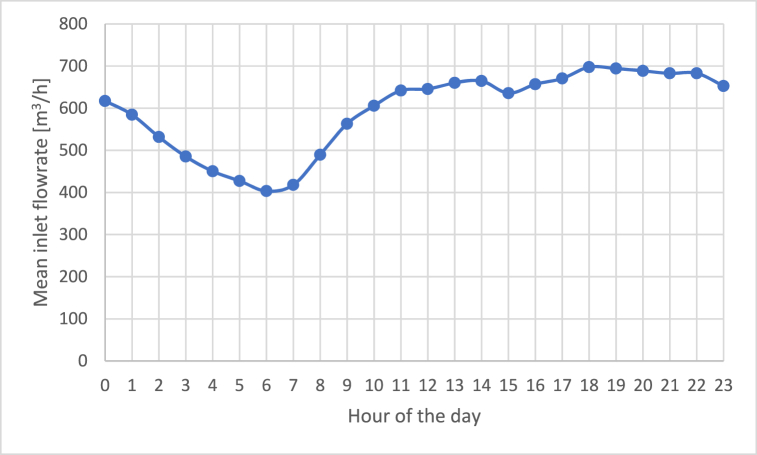
Daily trend of the mean inlet flowrate of the WWTP.

**Fig. 17:**
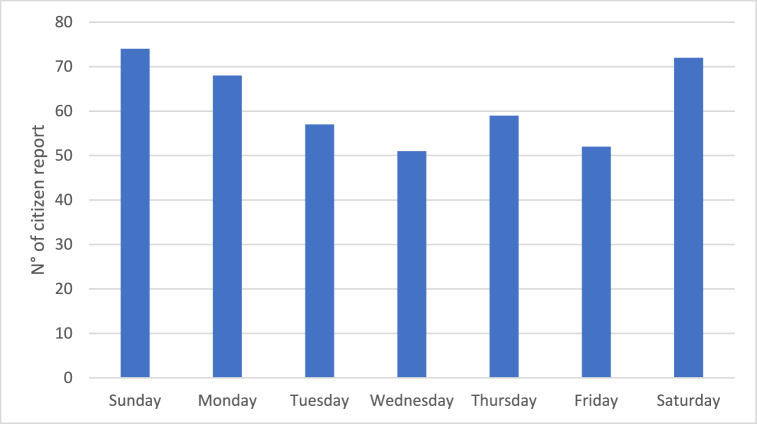
Analysis of the number of citizen reports for the different days of the week.

The graph shows that the exceeding of the NOR threshold is more frequent during the central hours of the day. More in detail, from 9 a.m. there is an increase of the number of exceedances, which grows until reaching a maximum from 11 a.m. to 1 p.m. Then, it gradually decreases returning back to moderate odour conditions. It should be noted that the exceedances of the IOR only happen in the central hours of the day, indicating particularly critical odour levels in those periods. This trend could partially be correlated with the inlet flowrate of wastewater at the WWTP during the day, since it is also characterized by an increasing trend in the early hour of the morning, followed by a stabilization during the central hours, and a decrease of the value in the late afternoon, as can be seen from Fig. 16. Fig. 17 shows the daily trend of the mean inlet flowrate at the WWTP relevant to the monitoring period.

#### 3.5.2 Correlation with citizens’ odour reports

For a deeper investigation of the occurrence of the odour events, the exceedances of the NOR as registered by the EN were compared with the odour reports by the citizens.

Citizens' reports were collected over a period of 12 months, 6 of which before, 3 during and 3 after the installation of the EN at the WWTP. During this period, a total of 433 reports were collected and analysed. All the citizens’ reports and not just the ones collected during the effective period of installation of the EN at the WWTP were used, with the purpose of relying on a more significant amount of data, thus obtaining a more representative outlook on the occurrence of odour events in the studied area.

Also in this case, first the distribution of odour reports over the different days of the week was investigated. The results of such analysis are reported in Fig. 17.

It can be observed that on Saturday, Sunday and Monday a higher number of complaints compared to the other days of the week was reported. This trend is very similar to the one observed for the NOR/alarm exceedances registered by the EN, showing a peak on the weekend and a general decreasing trend during the central days of the week.

As previously stated, this trend could be associated to the fact that the wastewaters reaching the plant over the weekend are more concentrated compared with the rest of the week. However, it should be highlighted that citizens’ reports could be also affected by some other factors, such as for instance the fact that during the weekend people are more likely to spend time at home or in public areas close to their houses, making that the number of odour perceptions may be increased.

**Fig. 18:**
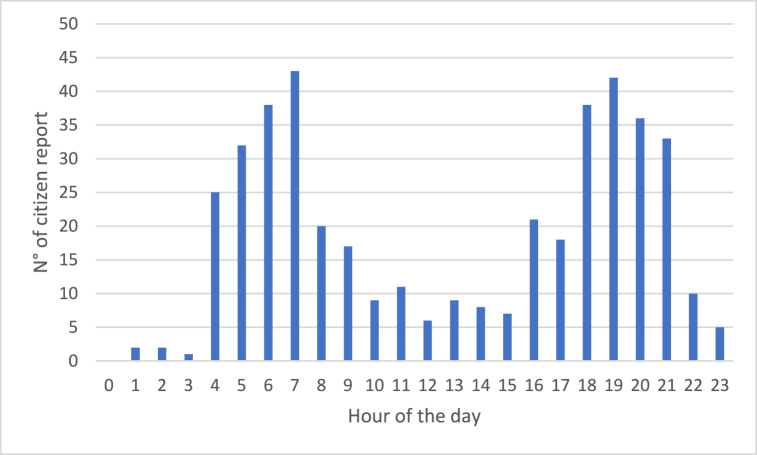
Analysis of the number of citizens' odour reports for the different hours of the day.

Moreover, we also analysed the daily trend of the number of citizens’ odour reports. The results, illustrated in Fig. 18, point out that citizens mostly complained the presence of odours attributable to the WWTP in the early morning from 4 a.m. to 7 a.m., and in the early evening from 6 p.m. to 9 p.m.

In this case, the information coming from the analysis of the citizens’ reports is not correlated with the trends obtained by the EN monitoring, which identified the central hours of the day as the most critical in terms of odour concentration inside the AT shed.

Still, this trend is not completely unexpected and can be justified considering the effects of atmospheric odour dispersion. As it is well known from the scientific literature and from the physics of the atmosphere, the most critical conditions for odour perceptions correspond to the so-called “thermal inversion” or “stable” conditions, in which the atmosphere is stably stratified with air temperature, increasing with the height from the ground, hindering vertical mixing of the air, and thus “trapping” odours and other pollutants close to the ground [64]. These conditions are typical of the early morning and of the evening (sunset). This behaviour can be visualized by applying an odour dispersion model to an odour source having a constant odour emission rate: the highest impact is typically predicted during these hours of the day [64]. Indeed, even though the EN shows that higher odour concentrations are emitted during the central hours of the day, the distribution of the odour complaints, showing an increase in the early morning and in the evening instead, proves that odour perception at receptors is strictly related to the mechanism of odour emission diffusion into the atmosphere and thus to the meteorological conditions.

Physically, the phenomenon could be explained considering that the odours emitted during the central hours of the day, typically characterized by so-called thermal instability and consequent mixing of the atmosphere, are dispersed vertically over the plant. However, as soon as the meteorological conditions change, and the mixing height of the atmospheric boundary layer collapses due to thermal inversion [65], the odours dispersed vertically during the day also collapse, causing a sudden increase in the concentration, which is then perceived by the citizens with a delay of few hours compared to the emission peak.

However, also in this case, when looking at citizens’ reports, other factors should be considered. As already observed, the hours during which most of the reports have been collected coincide with the moments during which people wake up and go out for work (morning) or return at home after work (evening), thus increasing the potential exposure to odour emissions and thus their perception.

Moreover, the presence of alternative odour sources was not considered here. We assumed that the complaints by the citizens are related to odours emitted by the WWTP based on the previous studies carried out in the area, but the presence of other sources causing such complaints cannot be completely excluded.

What emerges from these results is that for carrying out an analysis aimed at understanding and possibly identify the causes of nuisance perceived by the population in such a complex case, it is not sufficient to rely solely on citizens' reports nor on the results of olfactometric analyses carried out discontinuously at the source, but it is necessary to introduce a system for the direct and continuous monitoring of the emission under investigation. As an example, in this case, if relying only on the citizens' reports, we would have identified the odour peaks as happening during the early hours of the morning or in the evening. However, thanks to the continuous analysis carried out by the EN directly on the most critical odour source (in this case, the AT of the WWTP), it was possible to identify that such odour peaks are mainly generated during the central hours of the day, whereas citizens’ odour perceptions are strictly related to the way the odours generated during the central hours of the day are dispersed into the atmosphere. On the other hand, dynamic olfactometry, which is the reference method for quantifying odour concentration, is a discontinuous technique. Therefore, besides having required the withdrawal of a huge number of gas samples, it would not have allowed to precisely spot the occurrence of the odour peaks nor their frequency and distribution over time.

Conversely, the approach here proposed required the withdrawal of a relatively low number of samples to be analysed by dynamic olfactometry for the training of the IOMS, which then allowed to have a continuous and real-time estimation of the odour concentration directly on the emission. This further enabled the prompt identification of the anomalous odour peaks with the consequent targeted withdrawal of samples during these events.

## 4 Conclusions

This paper proposes a novel approach for implementing a multi-sensor system (“electronic nose”, EN) for the real-time identification of anomalous odour events related to variable and unknown compositions of wastewaters at the inlet of a WWTP. The EN was trained in order to identify the odour peaks and simultaneously allow the targeted withdrawal of gas and liquid samples to be further analysed for a chemical characterization to investigate the causes of such peaks. The introduction of this new methodology, involving the implementation of a specific classification model to detect deviations from a reference condition, differs from the more traditional approaches used for training EN for environmental odour monitoring. The development of such a novel approach was deemed necessary because of the undefinable number of different odour classes potentially occurring at the source under investigation (besides civil wastewaters, the plant receives the discharges from over 40 industries of different types), making it impossible to neither train the EN to recognize each one of them nor to use a single regression model for estimating the odour concentration.

More in detail, the proposed approach consists in the implementation of a one-class SVM aimed to the definition of a “Normal Operating Region” (NOR) representative of “normal” odour emissions (i.e., unlikely to cause nuisance in the population) able to identify “anomalous” deviations from it. The method developed proved its capability to identify the occurrence of anomalous odour events on the WWTP arrival tank with an accuracy of 90% (CI_95%_ 55–100%) on external validation data.

The EN outputs obtained during the monitoring phase, which lasted for about 50 days, were used for critically investigating the trends of the odour peaks. The results obtained highlighted that the odour peaks identified by the EN occurred mainly on Sundays and Mondays, and during the central hours of the day, i.e. from 9 a.m. to 1 p.m.

Citizens’ odour reports were also analysed, showing an increasing trend during weekends. This could be explained considering that the wastewaters reaching the plant over weekends may be more concentrated because of the weekly interruption of the activity of smaller industries discharging less odorous wastewaters.

Concerning the analysis on an hourly basis, citizens’ complaints turned out to be more frequent in the early morning and early evening, differently from the odour peaks detected by the EN, which are concentrated in the central hours of the day. This could be due to the fact that the atmospheric conditions that are typical of the early morning and early evening are particularly unfavourable for odour dispersion, thus increasing the probability that they are perceived outside of the plant. Indeed, the phenomenon of so-called “thermal inversion” could lead to a sort of “collapse” of the odours dispersed vertically during the day, increasing their concentration above the perception threshold, with a temporal shift compared to the emission peak that happens during the central hours of the day.

The main result of this paper is related to the proof that a properly trained EN is able to detect anomalous odour emissions in real-time and quantify the odour concentration. This way, it can serve as a very useful plant management tool for investigating the causes of such anomalous odour conditions in a complex scenario, such as the one here examined, where the odours emitted are characterized by an extremely variable and unpredictable chemical composition.

Despite the encouraging results here reported, it is important to also highlight the limitations of this study. One major limitation is the relatively low number of samples used for the validation of the model, which had to be limited due to budget restrictions for olfactometric analyses, which are very expensive. Moreover, it should be considered that the study only refers to a limited time period of approximately 4 months, during which no sensor loss of sensitivity or drift has been observed. However, it is legitimate to hypothesize that for long-time monitoring, such effects should be considered and possibly some drift mitigation strategies would need to be implemented, as well. As a final consideration, future work could focus on the extraction of other possible information from the continuous sensor signal timeseries, without relying solely on the averaged sensors response.

Such investigations may help improving the reliability of this methodology and make it applicable also to other situations. Indeed, the logic of training an instrument to recognize deviation from a target “reference condition” could find application in other scenarios, such as for instance process monitoring or quality control.

## Data availability statement

The data that has been used is confidential.

## Acknowledgements

This paper has been conceived and produced within the MUSA – Multilayered Urban Sustainability Action – project, funded by the European Union – NextGenerationEU, under the National Recovery and Resilience Plan (NRRP) Mission 4 Component 2 Investment Line 1.5: Strenghtening of research structures and creation of R&D “innovation ecosystems”, set up of “territorial leaders in R&D.

## CRediT authorship contribution statement

**Stefano Prudenza:** Writing – review & editing, Writing – original draft, Software, Investigation, Data curation. **Carmen Bax:** Writing – review & editing, Supervision, Data curation, Conceptualization. **Laura Capelli:** Writing – review & editing, Supervision, Project administration.

## Declaration of competing interest

The authors declare that they have no known competing financial interests or personal relationships that could have appeared to influence the work reported in this paper.
